# Behavioral Responses to Uncertainty in Weight-Restored Anorexia Nervosa – Preliminary Results

**DOI:** 10.3389/fpsyg.2019.02492

**Published:** 2019-11-05

**Authors:** Mayron Piccolo, Gabriella Franca Milos, Sena Bluemel, Sonja Schumacher, Christoph Mueller-Pfeiffer, Michael Fried, Monique Ernst, Chantal Martin-Soelch

**Affiliations:** ^1^Unit of Clinical and Health Psychology, University of Fribourg, Fribourg, Switzerland; ^2^Department of Consultation-Liaison-Psychiatry and Psychosomatic Medicine, University Hospital Zurich, Zurich, Switzerland; ^3^Division of Gastroenterology and Hepatology, University Hospital Zurich, Zurich, Switzerland; ^4^Zurich Center for Integrative Human Physiology, Zurich, Switzerland; ^5^Section on Neurobiology of Fear and Anxiety, National Institute of Mental Health (NIMH), Bethesda, MD, United States

**Keywords:** anorexia nervosa, intolerance of uncertainty, weight-restoration, longitudinal, eating disorders, remission

## Abstract

Impaired decision-making under conditions of uncertainty seems to contribute to the expression and maintenance of anorexia nervosa (AN), but it is not clear whether this impairment is a disease state that would remit with treatment, or a persisting trait in patients with AN. To examine this question, a longitudinal study was conducted in 12 female inpatients with AN (age *M* = 22.2, SE = 1.36), before (Time-1) and after reaching a body mass index of >17.5 kg/m^2^ (Time-2). Intolerance of uncertainty (IU) was assessed via a decision-making task, the wheel of fortune (WOF). Weight gain at Time-2 was accompanied with significant changes in uncertainty-related performance compared to Time-1 [(Time × Uncertainty), *p* < 0.05]. At Time-1, reaction times (RTs) varied in function of uncertainty, while at Time-2, uncertainty did not modulate RTs. These findings support a change in decision-making under uncertainty with successful weight-rehabilitation in AN. While IU was present in underweight patients, it became non-significant after weight restoration.

## Introduction

The mechanisms underlying anorexia nervosa (AN), a life-threatening condition, are still unclear (American Psychiatric Association, 2013). Impaired decision-making under conditions of uncertainty seems to contribute to the expression and maintenance of AN (Brown et al., 2017). In that sense, intolerance of uncertainty (IU) has become an important theme in eating disorders and other types of mental disorders (Brown et al., 2017). In the framework of our study, IU was previously operationalized in the context of decision making as the inclination for “certain” vs. “uncertain” outcome (Piccolo et al., 2019).

Decision-making under uncertainty and IU have been studied both using self-report and behavioral measures (Jacoby et al., 2014; Kesby et al., 2017; Piccolo et al., 2019). In terms of self-report, IU has been mostly measured by using self-reported data collected by the Intolerance of Uncertainty Scale (IUS), in its short (Carleton et al., 2007) and longer (Freeston et al., 1994) versions, which identified two main IU factors have been described: (1) a desire for predictability, also known as prospective IU, and (2) a sort of paralysis in the face of uncertainty, also known as inhibitory IU (Berenbaum et al., 2008). Desire for predictability is mostly associated with the anticipation of uncertain situations, while uncertainty paralysis represents a freezing behavior in the face of uncertainty (Kesby et al., 2017). These questionnaires were created to reflect both prospective and inhibitory IU (Berenbaum et al., 2008). In AN, responses to these questionnaires have been linked to the aversion of the inability to control outcomes (Sternheim et al., 2011a), which could be linked to a prospective IU. In addition, IU severity was found to be positively correlated with depression symptoms in major depression (McEvoy and Mahoney, 2011) and in AN (Abbate-Daga et al., 2015). Specifically to AN, subjective IU has been shown to correlate with cardinal features of the disorder, including *drive for thinness* and *body* dissatisfaction (Brown et al., 2017).

While mostly measured with self-report questionnaires, responses to uncertainty have recently been examined by using experimental tasks (Sternheim et al., 2011b; Jacoby et al., 2014; Piccolo et al., 2019). In AN, results are mixed, with one study showing difference in behavioral responses (Piccolo et al., 2019), and the other not (Sternheim et al., 2011b). The difference might be due to the nature of the tasks used, with one being a data-gathering task (Sternheim et al., 2011b), which might mostly relate to the prospective factor of IU, and the other, a decision-making task (Piccolo et al., 2019), mostly related to inhibitory IU.

More recently, we have assessed decision-making in patients with AN by using the well-validated wheel of fortune (WOF) task that probes decision-making under varied uncertainty levels (Ernst et al., 2004). Reaction time (RT) served as a measure of sensitivity to uncertainty, with longer RT signaling greater sensitivity to uncertainty (i.e., IU). Findings revealed longer RTs to choices with “uncertain” outcomes in patients with AN compared to healthy controls (Piccolo et al., 2019). The present behavioral study tests whether AN-related behavioral IU, defined as delayed decision-making under uncertainty, resolved after weight-rehabilitation treatment. As a potential vulnerability factor, behavioral IU would be expected to remain present after weight restoration, but as a symptomatic expression of AN, IU would be expected to decrease along with other symptoms.

## Methods

A longitudinal study was conducted in 12 female inpatients with severe underweight (mean Body-Mass Index, BMI, 14.6 kg/m^2^, see Table 1), carrying the DSM-IV-R diagnosis of AN (American Psychiatric Association, 2000). Initially, 24 participants were recruited, with only 12 reaching expected BMI at the end of treatment. Testing was conducted at two time points, before treatment and after weight-restoration, with patients who had reached a minimum of 17.5 kg/m^2^. This weight was considered because a BMI of 17 kg/m^2^ represents the threshold for an AN diagnosis (American Psychiatric Association, 2000). IU was assessed via measures of RTs to a decision-making task, the WOF, which assesses decision patterns under uncertainty (Ernst et al., 2004; Piccolo et al., 2019). The WOF consists of three types of two-slice wheels. Each of the three wheels displays unique probabilities (i.e., degrees of certainty) of a monetary gain or loss. These three types of wheel consist of (1) a 50% chance of loss vs. a 50% chance of gain (50/50 trial, maximum uncertainty, *n* = 24), (2) a 30% chance of gain vs. a 70% chance of loss (30/70 trial, *n* = 16), and (3) a 10% chance of gain vs. a 90 chance of loss (10/90 trial, *n* = 22). Gain magnitude is weighed according to probability, such that the amount of gain is pitted against the probability of the outcome. As such, the higher reward is associated with the lower probability. For instance, the 10/90 wheel features the options of 90% chance of winning $ 1 and 10% chance of winning $ 4. The 50/50 wheel consists of equal chances of winning the same reward ($ 0.50) (Ernst et al., 2004). RTs were used as a variable because they reflect the time taken to make a decision, and has been previously related to IU (Piccolo et al., 2019). At the end of the experiment, participants received the amount of money they had won during the task.

**TABLE 1:** Demographic and clinical scores of AN participants.

**Measure (mean ± SE)**	**Time-1**	**Time-2**	***t***	***p*-value**
BMI kg/m^2^	14.6 ± 0.2	18.1 ± 0.2	−9.930	0.000
(range)	(13.2–16.0)	(17.6–19.3)		
Age	22.2 ± 1.3	22.2 ± 1.3	−	−
(range)	(17–32)	(17–32)		
BDI	29.1 ± 3.0	13.2 ± 2.7	5.634	0.000
STAI trait	54.3 ± 3.2		−	−
STAI state	49.1 ± 4.0	46.0 ± 5.0	0.962	0.361

*Body mass index - BMI-, depression level assessed by the Beck Depression Inventory (BDI), and anxiety level assessed by the State-Trait Anxiety Inventory (STAI) after admission (Time-1) and after weight rehabilitation (Time-2) in inpatients with anorexia nervosa. Comparisons between Time-1 and Time-2 were performed with *t*-tests.*

Because of the high comorbidity between AN and depression and anxiety symptoms (Woodside and Staab, 2006), participants were screened for depression using the Beck Depression Inventory (BDI) (Hautzinger et al., 1994), and anxiety symptoms using the State-Trait Anxiety Inventory (STAI) (Laux et al., 1981). Data at admission (Time-1) were collected during the orientation phase of the inpatient stay, a period for psychiatric and psychosomatic symptoms stabilization. Toward the end of an intensive multimodal treatment, approximately 3 months after the beginning of the treatment (Time-2), the same data collection done at admission was repeated. Because trait anxiety refers mostly to stable anxiety proneness (Laux et al., 1981), trait anxiety was only measured in Time-1.

The objective of the treatment is to reduce pathological eating behaviors, improve body-weight, and treat concomitant psychiatric co-morbidities. The treatment components included medical examination and treatment; structured balanced-meals, meals debriefings and weight monitoring; individual psychotherapy including meals diary and nutritional counseling; meals planning and cooking training; group psychotherapy and psychodidactic group meetings; body perception therapy and gymnastic; art therapy; social skills training; review of daily activity structure and job training; family meetings; and social counseling. Finally, the psychosocial situation of the patients was assessed and patients received adequate support to facilitate changes before discharge, in an attempt to stabilize and maintain improvements achieved during therapy.

The study was approved by the Ethics Committee from the University of Zurich (KEK-ZH-No 2009-0115/1) and registered on ClinicalTrials.gov (NCT00946816). All participants provided written informed consent.

Analyses were conducted on RT (Piccolo et al., 2019). RT was examined using linear mixed models with maximum likelihood estimation. Full factorial models were fitted including the time of data collection (Time: Time-1, Time-2) and the probability (50/50, 30/70, and 10/90 trials) as fixed factors. A diagonal covariance structure with Bonferroni *post hoc* correction for pairwise comparisons was implemented for the repeated observations. Depression and anxiety scores were compared by paired-sample *t*-test. Statistical analyses were performed using IBM SPSS Statistics 25 (IBM Corp., Armonk, NY, United States).

## Results

Weight-restoration was demonstrated with the improvement of BMI (BMI_Time__1_ *M* = 14.6 kg/m^2^, SE = 0.2; BMI_Time__2_ = 18.1 kg/m^2^, SE = 0.2; *p* < 0.001) at Time-2 (see Table 1). The Time × Probability interaction on RT was significant (F_(__2__.__2886__.__92__)_ = 3.398, *p* < 0.05). At Time-1, RT to 50/50 conditions (*M* = 3203.60, SE = 412.08) was slower compared to the other conditions (*M*_(__30__/__70__)_ = 2514.423, SE = 417.53; *M*_(__10__/__90__)_ = 2556.413, SE = 413.294), while at Time-2, there was no statistical RT difference between probabilities (*M*_(__50__/__50__)_ = 2786.33, SE = 412.01; *M*_(__30__/__70__)_ = 2532.14, SE = 417.82; *M*_(__10__/__90__)_ = 2591.78, SE = 413.15).

**FIGURE 1:**
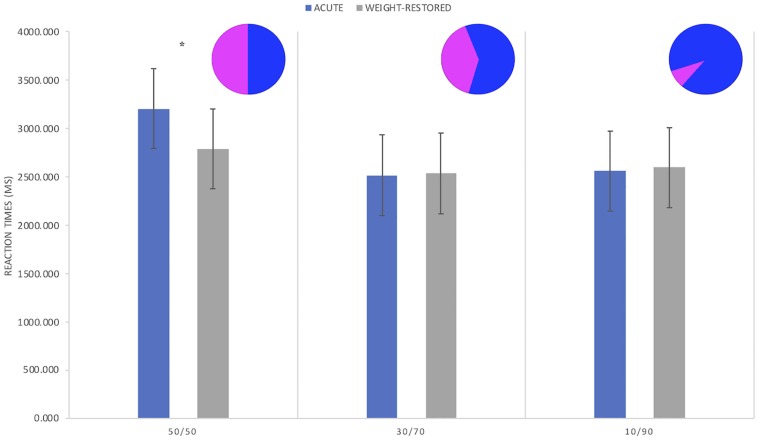
Reaction times (RT) in patients with anorexia nervosa during acute (Time-1) and after weight-recovery phase (Time-2). In Time-1, participants significantly had slower RTs to trials involving 50/50 probabilities in comparison to the other conditions, and to the same condition after recovery. No significant differences were found for the other probabilities (^∗^*p* < 0.05).

In addition, as expected, a main effect of Probability was present (*F*_(__2__.__2886__.__92__)_ = 13.511, *p* < 0.001), with longer RT in the 50/50 (*M* = 2994.96, SE = 398.56) when compared to the other conditions (*M*_(__30__/__70__)_ = 2523.28, SE = 401.52; *M*_(__10__/__90__)_ = 2574.10, SE = 399.17) across the whole sample. Depression symptoms significantly improved after treatment (Time-1 *M* = 29.1, SE = 3.0; Time-2 *M* = 13.2, SE = 2.7; *p* < 0.001). State anxiety scores remained unchanged (Time-1 *M* = 49.1, SE = 4.0; Time-2 *M* = 46.5, SE = 5.0; *p* = 0.361) (see Figure 1).

## Discussion

This study tested whether behavioral IU remains present after weight-restoration in patients with AN. While impaired decision-making under uncertainty was present in symptomatic patients, it became non-significant after weight restoration. These findings suggest that impaired decision-making under uncertainty might be a behavioral expression of the underweight condition in AN, which does not remain as a sequel after successful weight-treatment.

Shorter RTs to uncertain conditions could represent the behavioral aspect associated to inhibitory IU, and are in line with a previous study that reported that IU scores (measured via questionnaires) were less severe in women recovered from AN compared to symptomatic patients (McFadden et al., 2014). The task used in the present study present uncertain options requiring actions from participants. These actions in the face of uncertainty, if delayed (longer RT), could be characterized along IUS items such as *“when it’s time to act, uncertainty paralyzes me,” “when I’m uncertain, I can’t function well,”* and *“the smallest doubt can stop me from acting”* (Carleton et al., 2007). However, when compared to other behavioral experiments that tested whether RTs to uncertainty was different in AN (Sternheim et al., 2011b; Jacoby et al., 2014), our study found different results. The task used in the previous studies consisted of jars containing different proportions of colored beads. Participants had to decide which jars the beads were being taken out from, and could choose as many beads as they thought necessary to come to a final decision. That behavioral study used objective measures employed by a different task, a data-gathering one, based on the need of anxious patients to gather more information before deciding among uncertain options. In other words, more bits of information decrease uncertainty. This could account for the difference between results. Further studies, using different measures of anxiety are required for clarifying this aspect.

The results of IU could also be related to the improvement of depression during the treatment, since depression scores and IU were found to be correlated in eating disorders (Sternheim et al., 2017). Indeed, IU and depression have long been associated, with IU mediating how reward is perceived in depressed patients (Nelson et al., 2014). Although depression may blunt cognitive responses such as RTs (Beaujean et al., 2013), this is likely not the case here, since responses to other categories would also reflect this. Furthermore, in comparison to normal-weight controls, only RTs to 50/50 categories were different in underweight patients with AN (Piccolo et al., 2019).

Of note, subjective scores of anxiety did not decrease after weight-gain treatment. Research regarding subjective anxiety is inconclusive regarding its role in AN, with some studies reporting improvements and others observing no changes after intervention (Kezelman et al., 2015). The presence of anxiety in weight-rehabilitated AN patients may be associated with the risk of relapses (Grilo et al., 2012), and should be addressed in AN treatment.

Future studies are warranted to validate and extend these findings. For example, the generalizability of the findings to other types of reward is important to assess, including primary rewards, such as food. In addition, the role of context (e.g., a feeling of safety vs. threat) in which decision-making under uncertainty is measured needs to be elucidated. Furthermore, the potential role of IU in maintaining disease-related behaviors needs further investigation, especially in fully recovered patients (i.e., remitted, and not only weight-restored).

This study presents some limitations. First, the sample size was small. Second, the methodology allowed for evaluation of behavioral and cognitive aspects related to IU, remaining the question of whether this accounts also for the affective aspect connected to IU as well. Also, we only measured IU in the context of decision-making, and have not explored how our measures are associated to responses to the questionnaires evaluating IU. Additionally, STAI-trait was only measured once, while other studies have evaluated it in different time points. In addition, although this task has been previously used as a pre- and post-intervention measure, and no learning effect was indicated (Dichter et al., 2009), responses could be related to an habituation to the task. It is likely though that, in the case of habituation, an effect would also have been seen to other categories. Finally, we could not control for symptoms such as amenorrhea, since some participants were taking medication related to it.

To the best of our knowledge, this study is the first to report behavioral data on decision-making under uncertainty, and improvement of decision-making under uncertainty in patients with AN after weight restoration. These findings might have implications for the treatment of AN. For instance, evaluation of IU, besides weight, during treatment in AN might help keep track of the efficacy of the treatment, and thus redirect intervention if needed. Furthermore, assessing IU in fully recovered (i.e., remitted) patients could provide further knowledge on the relationship between IU and AN, since here, data were collected in weight-restored patients.

## Data Availability Statement

The datasets generated for this study are available on request to the corresponding author.

## Ethics Statement

The studies involving human participants were reviewed and approved by the Ethics Committee from the University of Zurich. Written informed consent to participate in this study was provided by the participants’ legal guardian/next of kin.

## Author Contributions

MP and ME performed data analysis, interpretation, and wrote the manuscript. GM, SB, MF, and CM-S developed the study protocol, performed the study, obtained the funding, and critically revised the manuscript. SS and CM-P performed data analysis and critically revised the manuscript.

## Conflict of Interest

The authors declare that the research was conducted in the absence of any commercial or financial relationships that could be construed as a potential conflict of interest.
